# Real-Time PCR based test for the early diagnosis of *Haplosporidium pinnae* affecting fan mussel *Pinna nobilis*

**DOI:** 10.1371/journal.pone.0212028

**Published:** 2019-02-22

**Authors:** Monserrat López-Sanmartín, Gaetano Catanese, Amalia Grau, José María Valencia, Jose Rafa García-March, José Ignacio Navas

**Affiliations:** 1 IFAPA Agua del Pino, Cartaya, Huelva, Spain; 2 LIMIA Govern de les Illes Balears, Port d’Andratx, Mallorca, Spain; 3 INAGEA (INIA-CAIB-UIB) Ctra, Valldemossa, Edifici Guillem Colom Casasnoves, Palma de Mallorca, Illes Balears, Spain; 4 IMEDMAR-UCV Universidad Católica de Valencia, Calpe, Spain; University of Helsinki, FINLAND

## Abstract

Noble pen shell or fan mussel, *Pinna nobilis* Linnaeus (1758), protected since 1992, was incorporated into the Spanish Catalogue of Threatened Species (Category: Vulnerable, Royal Decree 139/2011). The status is presently in the process of being catalogued as critically endangered, pending approval by Spanish Government (https://www.mapama.gob.es/es/biodiversidad/participacion-publica/Borrador_OM_situacion_critica.aspx). The International Union for the Conservation of Nature (IUCN) alerted the countries of the Mediterranean basin to the “emergent situation” due to serious mortality events suffered by the fan mussel, putting it in serious risk of extinction. Thus, emergency actions have been implemented by Spanish authorities in which several research institutes from all over the country are involved. The parasite, *Haplosporidium pinnae*, was recently characterized by histology, TEM, SEM and molecular biology techniques and it was considered responsible for the mass mortality of *P*. *nobilis* in the Mediterranean Sea. In this context, the aim of this study has been to develop species-specific quantitative PCR (qPCR) protocol carrying out a fast, specific and effective molecular diagnose of *H*. *pinnae*. In this sense, the detection limit for qPCR was equal to 30 copies of SSU rDNA / ng of DNA using plasmid alone and when 100ng DNA of non-infected oyster were added. The qPCR assay revealed that 94% of the 32 analysed mantle tissues of fan mussel were infected by *H*. *pinnae*, showing a high sensitivity and specificity for its detection (100% if we don't consider negative and too much degraded samples). This technique will allow us to make quicker follow-ups of the disease, allowing us to get a better understanding of its evolution in order to help in the rescue of *P*. *nobilis* populations

## Introduction

The fan mussel (*Pinna nobilis* Linneaus, 1758) is an emblematic marine bivalve endemic to the Mediterranean Sea. It is the largest in this area and one the largest bivalves in the world. By the late twentieth century, the populations of fan mussels decreased associated with years of human activity [1–2]. As a consequence, *P*. *nobilis* have been protected since 1992 by the Annex II of the Barcelona Convention (SPA/BD Protocol 1995), Annex IV of the EU Habitats Directive (EU Habitats Directive 2007), and Spanish Catalogue of Threatened Species (Category: Vulnerable, Royal Decree 139/2011) [2].

Recently, a mortality event of *P*. *nobilis*, caused by a haplosporidian protozoan parasite, has been reported in western Mediterranean [3]. In autumn 2016, worries of high mortality rates reaching up to 100% in the central and southernmost coasts of the Spanish Mediterranean Sea were reported [2]. Fan mussels showed non-specific warning signs of illness: mantle retraction, gaping, slow closing, slow response to touch and reopening of the valves after a short time, resulting finally in death [2]. The characterization of the parasite by histology, TEM, SEM and molecular biology techniques confirmed that the causal agent was a new species included in the phylum Haplosporida. The name *Haplosporidium pinnae* has been suggested as this new pathogen very likely is responsible for the mass mortality of *P*. *nobilis* in Mediterranean Sea [4].

In summer 2017, other Spanish and Mediterranean areas reported on the critical situation in the *P*. *nobilis* population mortality. The International Union for the Conservation of Nature (IUCN) alerted the countries of the Mediterranean basin of the "emergency situation" due to the fan mussel mortality caused by the parasite and implemented different actions for the principal purpose of the conservation of this species with extinction risk (www.iucn.org/news/mediterranean/201807/emergency-situation-pen-shells-mediterranean). As a consequence, Spanish authorities are changing the status of the species from vulnerable to critically endangered (https://www.mapama.gob.es/es/biodiversidad/participacion-publica/Borrador_OM_situacion_critica.aspx) and approved an emergency action in October 2017, for moving and maintaining 215 *P*. *nobilis* individuals in 5 Spanish aquaculture facilities, including the IFAPA Agua del Pino (Southeast of Spain) Research Centre.

The rescue measures carried out are incomplete without parallel actions that contribute to the survival of the fan mussel *in situ*. In fact, developing a rapid and effective diagnostic method could make an important contribution to the research and control of *H*. *pinnae* disease, so that the scientific community would be able to design actions protocols to improve the critical situation of *P*. *nobilis*.

This type of actions will allow monitoring, and effectively begin emergency protocols to protect susceptible fan mussel of the infection in the natural environment [5].

In fact, the fan mussels rescued from "Cala de Portlligat" (Cap de Creus, Girona, NE Spain) and maintained in captivity in IFAPA Agua del Pino facilities, were initially considered as non-affected by *H*. *pinnae*. Although they were maintained in quarantine conditions with temperature, salinity and food controlled daily, after several weeks some specimens showed unspecific warning signs of illness, resulting finally in death. Therefore, specific, rapid and sensitive diagnosis is critical to increase the effectiveness of rescue programs designed to maintain fan mussels in captivity.

Molecular biology techniques are increasingly used in pathogen diagnostics, since PCR is highly specific and sensitive compared with other diagnostic tests. Nowadays, real-time PCR is a commonly used technique for the detection, quantification and sorting of different pathogen agents in the areas of clinical and veterinary diagnostics [6]. The real-time PCR (also denoted as quantitative PCR or qPCR), unlike conventional PCR, measures fluorescence after each cycle and the intensity of the fluorescent signal reflects the momentary amount of DNA amplicons in the sample at a specific time. The main advantage of this approach is the sensitivity, because it can detect down to just a few molecules of target DNA. Semi-quantitative results can also be provided by qPCR using controls such as a reference material [6].

Our study provides a useful tool to assess the health status of *P*. *nobilis* by the development of a fast, specific and effective diagnostic procedure to detect *H*. *pinnae* in fan mussel specimens. Species-specific conventional PCR (cPCR) techniques were used to diagnose the presence / absence of *H*. *pinnae* and then a quantitative PCR (qPCR) was focused to reveal the *H*. *pinnae* parasitic load. The qPCR protocol, involving a one-step procedure, is reliable, sensitive, and quick to perform, enabling it to be also applied as potential diagnostic technique for different marine organisms and environmental samples in the future studies to identify if *H*. *pinnae* are affecting other marine organisms.

## Materials and methods

### Sample collection and genetic DNA extraction

Due to status as an endangered and protected species, sampling and transport of *P*. *nobilis* was carried out under permission of regional and national Authorities.

The samples were collected from recently dead specimens between December 2017 and May 2018 both from wild adult fan mussel living in Port d’Andratx (Mallorca, Spain) and from adults held at IFAPA Agua del Pino (Huelva, Spain) facilities (origin Portlligat, Girona). Duplicate portions of the mantle (n = 31) and adductor muscle (n = 1) were collected from each specimen. Molecular diagnosis of these samples was carried out in two different laboratories: at IFAPA Agua del Pino and at LIMIA (Mallorca, Spain). The characteristics of each fan mussel sample analysed in this study are shown in **Table 1**.

**Table 1:** Sampling data of *Pinna nobilis* (n = 31) specimens analysed in this study.

Pen shell name	Date of death	Height (cm)	Width (cm)	Weight (g)
PN1	16/12/2017	49	23	229
PC*	-	-	-	-
PN2	15/02/2018	56	23	188.25
PN3	22/03/2018	57.5	21.5	196.05
PN4	23/03/2018	49	22	139.1
PN5	26/03/2018	50	21.5	150.8
PN6	28/03/2018	64.5	23	246.87
PN7	28/03/2018	64	22	194.81
PN8	06/04/2018	59.5	25	344.96
PN9	11/04/2018	53	22	24763
PN10	12/04/2018	54.5	21.5	149.96
PN11	13/04/2018	53.5	22	203.78
PN12	13/04/2018	66.5	25	199.55
PN13	16/04/2018	47.5	21	82.81
PN14	16/04/2018	57	22	152.16
PN15	16/04/2018	58	22	156.02
PN16	16/04/2018	46	21	193.26
PN17	23/04/2018	45	22	163.08
PN18	23/04/2018	56	23	155.63
PN19	25/04/2018	57	22	ND
PN20	03/05/2018	65	25	218.35
PN21	03/05/2018	50.5	21	204.2
PN22	04/05/2018	50.5	21.5	151.5
PN23	07/05/2018	64	23	187.21
PN24	07/05/2018	64	23	218.82
PN25	08/05/2018	49.5	19	173.07
PN26	21/05/2018	55	21.5	86.27
PN27	22/05/2018	49.5	20	164.54
PN28	23/05/2018	53	23.5	205.32
PN29	31/05/2018	41	18.5	103.7
PN30	31/05/2018	54	24	283.32

Mantle was the tissue sampled in every fan mussel and adductor muscle and mantle for PN27.

(*) Positive control from Port d’Andratx (Mallorca, Spain)

The tissues were stored in DMSO-EDTA-NaCl (20% DMSO, 0.25 M EDTA and 30mg/ml NaCl) or absolute ethanol and then maintained at 4°C [7]. Before DNA extraction, the tissues were cut and put in distilled water for 1 minute to account for rehydration and residue removal. The DNA extraction was carried out using the E.Z.N.A. Tissue DNA (OMEGA bio-tek) or Nucleospin Tissue (Macherey-Nagel) commercial kits, following the manufacturers' protocols. DNA quality and quantity were proved using a Nanodrop NC-100 spectrophotometer (Nanodrop Technologies). Final DNA concentrations were adjusted for each sample: until 100 ng/μl with elution buffer for conventional PCR and to 50 ng/μl with sterile deionized water for qPCR assays.

### Primer design

In this study, specific primers were designed for cPCR and qPCR amplifications, based on the 18S rDNA (SSU rDNA) gene sequence of *H*. *pinnae*, obtained from Catanese et al 2018 [4] (**Table 2**). To confirm specificity and that the amplifications discriminate between genera and species, the *H*. *pinnae* DNA sequence (LC338065) and the DNA sequences from GenBank of 15 different haplosporidian species were aligned using the programme Clustal W: *Haplosporidium costale* (KC578010.1); *Haplosporidium nelsoni* (AB080597.1); *Haplosporidium diporeiae* (KF378734.1); *Haplosporidium patagon* (KJ534587.1); *Haplosporidium raabei* (HQ176469.1); *Haplosporidium tuxtlensis* (JN368430.1); *Haplosporidium pickfordi* (AY452724.1); *Haplosporidium montforti* (DQ219484.1); *Haplosporidium lusitanicum* (AY449713.1)*; Minchinia tapetis* (AY449710.1); *Minchinia teredinis* (U20320.1); *Minchinia chitonis* (AY449711.1); *Bonamia perspora* (DQ356000.1); *Bonamia ostreae* (JN040831.1) and *Bonamia exitiosa* (JF831802.1). Subsequently, the primers were designed using the programme Primer 3 [8–9]. To diagnose the presence of *H*. *pinnae* in the samples, the primer pair HPNF3/HPNR3 was initially used as described by Catanese et al 2018 [4] and then specific pair of primers HpF/HpR and HpF3/HpR3 were designed to amplify fragments of 1409 bp and of 165 bp, respectively. These two fragments were used for comparative diagnostic between cPCR and qPCR assays and also for estimating the sensitivity of each of the different approaches (see below). A general control of PCR amplifications using 18SFr/18SRw primers was carried out for all samples to confirm the integrity of extracted DNA and to discard that some kind of inhibitor was present [10].

**Table 2:** Characteristics of the primers used for PCRs.

Primer	Sequence (5´- 3´)	Fragment size (bp)	Temperature annealing (°C)	Reference
18SFr	CGAGCAATAACAGGTCTGTG	200	50°C	Mauri et al 2011
18SRw	GGCAGGGACTTAATCAA			
HPNF3	CATTAGCATGGAATAATAAAACACGAC	600	55°C	Catanese et al 2018
HPNR3	GCGACGGCTATTTAGATGGCTGA			
HpF	GGTACGGAGAATCCGGGGTT	1409	55°C	This study
HpR	ACTTGTCCTTCCTCTAATAATAAGG			
HpF3	GCGGGCTTAGTTCAGGGG	165	60°C	This study
HpR3	ACTTGTCCTTCCTCTAATAATAAGG			

### Conventional PCR and sequencing to identify *Haplosporidium pinnae*

The conventional PCR assays [4] were performed in two Labs using different protocols:

- at IFAPA Lab: in a total volume of 25 μl containing genomic DNA (100 ng), 5 μl of 5x My Taq Red Reaction Buffer (Bioline), 0.4 μl of each primer (10 μM), 0.2 μl of My Taq Red DNA Polymerase (Bioline) and adjusted to 25 μl with sterile deionized water;
- at LIMIA Lab: in a total volume of 20 μl containing genomic DNA (100 ng), 0.4 μl of each primer (stock 20 μM), 10 μl of KAPA Taq ReadyMix PCR Kit (KapaBiosystems) and adjusted to 20 μl with sterile deionized water.

The primer pair and the annealing temperatures for each PCR are shown in **Table 2**. Positive samples for *H*. *pinnae* by conventional PCR were purified with illustra ExoProStar enzymatic PCR and Sequencing Clean-Up kit (GE Healthcare UK Limited) according to the manufacturer´s instructions. The sanger method sequencing was performed for all positive *H*. *pinnae* PCR amplifications by STABVIDA Service (Portugal). The chromatograms were analysed using the programme ChromasPro version 2.6.4 (Technelysium). Regions of similarity between edited and published biological sequences were compared using the BLAST-Basic Local Alignment Search Tool (https://www.ncbi.nlm.nih.gov/BLAST/).

### Sensitivity and specificity

To determine the analytical sensitivity of the conventional and quantitative PCR assays, two PCR products were amplified using HpF/HpR and HpF3/HpR3 pair primers. Then, those amplicons were ligated into vectors for cloning (pGEM-T vector systems; Promega) at 4°C overnight and inserted into *E*. *coli* One Shot Top 10F´ Chemically Competent Cells (Invitrogen Life Technologies). Transformed cells were screened by PCR using the same primers described above. Plasmid DNA of positive clones was purified using the PureLink Quick Plasmid Miniprep kit (Invitrogen). DNA quality and quantity were measured in the Nanodrop NC-100 spectrophotometer (Nanodrop Technologies).

The sensitivity of the PCR assays with HPNF3/HPNR3 and HpF3/HpR3 pairs of primers was analysed by serial dilutions 10-folds of the plasmid alone and mixed with 100 ng of fan mussel DNA covering from 300 million to < 3 *H*. *pinnae* plasmid copies. The specificity was analysed using DNA from cockles infected by *Marteilia cochillia*, clams infected by *Perkinsus olseni*, mussels infected by *M*. *refringens* and oyster infected by *Bonamia exitiosa*.

### Real-time PCR for quantitative detection of *Haplosporidium pinnae*

The qPCR assay for *H*. *pinnae* detection and quantification was carried out in duplicate using the species-specific primer pair (HpF3/HpR3) in a Mx3000P Thermocycler (Agilent). Amplification reactions were performed in a total volume of 10 μl comprising: 2 μl of genomic DNA (100 ng), 5 μl of PowerUp SYBR Green Master Mix (Applied Biosystems), 0.2 μM each specific primer and adjusted to 10μl with distilled water. Negative control (without DNA or with *H*. *pinnae* non-infected fan mussel DNA, previously confirmed by qPCR), positive controls (samples with positive cPCR amplification for *H*. *pinnae*) and standard curve (plasmid HpF3/HpR3) were included in each qPCR assay. After testing various annealing temperature, the final qPCR program was: 1 cycle for 2 min at 50°C; 1 cycle for 2 min at 95°C, 40 cycles of amplification at 95°C for 15 s, 60°C for 18 s and 72°C for 1 min and followed by a dissociation stage for determining the melt curve (Quantitative SYBR Green with dissociation curve). Two standard curves were calculated using serial dilutions of the plasmid and plasmid diluted with DNA of fan mussel; the efficiency (E) was from the slope of the standard curve following formula [11] E = 10^−1/slope^-1. Melting curve was generated with temperatures increments of 0.5°C s^-1^ starting at 60°C and ending at 95°C in order to ensure that a single PCR product was amplified for the primers dx.doi.org/10.17504/protocols.io.xmyfk7w. Amplification was also confirmed by electrophoresis on 2% agarose gels, in 1% TAE (Tris acetate EDTA buffer), stained with Red Safe (Nitron Biotechnology) and scanned in a GelDoc-It Imagen System Ultraviolet Transilluminator (BioImaging Systems). The protocol has been deposited in protocols.io, where has been assigned its own identified (dx.doi.org/10.17504/protocols.io.xe7fjhn)

#### Concordance analysis

To estimate the degree of agreement (concordance) between the PCR results performed on the same samples Cohen's Kappa coefficient was calculated [12]. When comparisons were made taking into account the intensity of the band in the agarose gel (-, +, ++, +++) the weighted Kappa was used [13].

To compare the diagnostic frequencies between assays, the Pearson Chi-square test was applied with Bonferroni correction to adjust the significance values. The significance limit for rejecting the null hypothesis was p <0.05. The statistical analyses were carried out using the IBM SPSS Statistics program (v.21).

## Results

A total of 32 samples from 31 fan mussels (31 mantle and 1 adductor muscle) were analysed in IFAPA laboratory and 30 samples from 29 fan mussels (29 mantle and 1 adductor muscle) in LIMIA laboratory using specific primers to target the SSU rDNA fragment of *H*. *pinnae*.

All the PCRs using the control of amplification generated amplicons with the exception of two samples (PN25 and PN30). The results of cPCRs using the primers HPNF3/HPNR3 showed that at least 20 of 29 mantle samples analysed in LIMIA laboratory were positive for *H*. *pinnae*. The same assay carried out in IFAPA showed that at least 15 of 31 mantle samples were positive for *H*. *pinnae*. When the diagnostic was carried out with HpF3/HpR3 primers, positive amplifications were 21/31. Nine samples (PN3, PN7, PN9, PN23, PN25, PN26, PN27, PN29 and PN30) resulted with negative cPCR amplification, or at least uncertain, using the different pair of primers for both laboratories. The results observed in IFAPA in five samples following the same conditions (PN1, PN21, PN22, PN24 and PN2) showed negative amplification with HPNF3/HPNR3 but positive with HpF3/HpR3. The cPCR results are summarised in the **Table 3**. The agreement (concordance) between laboratories was substantial with the primer pair HPNF3/HPNR3 (weighted Kappa = 0.630) and almost perfect (weighted Kappa = 0.07) comparing the results of LIMIA-HPNF3/HPNR3 and IFAPA- HpF3/HpR3 primers [14].

**Table 3:** Summary of the results obtained for the diagnosis of *H*. *pinnae* by conventional PCR (cPCR) in LIMIA and IFAPA and by qPCR in IFAPA.

Pen shell name	LIMIA	IFAPA		
	cPCR	cPCR		qPCR
	HPNF3 / HPNR3	HPNF3 / HPNR3	HpF3 / HpR3	HpF3 / HpR3 (DNA copies of *H*. *pinnae* / ng DNA)
PN1	ND	-	+	1.5E+02
PC*	+++	+++	+++	7.1E+07
PN2	ND	-	-	3.1E+01
PN3	-	-	-	-
PN4	+++	+++	++	3.1E+04
PN5	+++	+++	+++	3.2E+03
PN6	+++	+++	+++	3.5E+02
PN7	-	-	-	-
PN8	+++	+++	++	6.4E+04
PN9	-	-	-	2.3E+01
PN10	++	+++	+++	1.3E+03
PN11	++	+++	+++	9.9E+05
PN12	+++	+++	+++	3.4E+05
PN13	+++	+++	+++	3.7E+05
PN14	+++	+++	+++	8.5E+05
PN15	++	++	++	1.3E+01
PN16	+++	+++	+++	2.3E+05
PN17	++	+++	++	4.8E+05
PN18	+++	+++	+++	2.2E+04
PN19	+++	+++	+++	4.6E+04
PN20	+++	-	-	3.6E+04
PN21	+++	-	++	1.3E+03
PN22	+++	-	++	7.9E+02
PN23	-	-	-	2.0E+00
PN24	+++	-	++	1.7E+03
PN25	-	-	-	< 1 copy
PN26	-	-	-	8.2E+00
PN26-AM	+	+	+	2.0E+01
PN27	-	-	-	6.3E+00
PN28	+++	-	+	6.6E+00
PN29	- ?	-	-	3.8E+01
PN30	- ?	-	-	< 1 copy

(ND) undetermined, (-) negative, (- ?) negative uncertain, (+) positive weak, (++) positive, (+++) clearly positive, (AM) adductor muscle

The sequences obtained from all positive cPCR samples using HPNF3/HPNR3 primers in IFAPA showed 100% of similarity with the published sequence of *H*. *pinnae* [4] (Accession number MK142774 to MK142779). No amplification was showed when we used these primers with DNA from cockles, clams, mussels and oysters infected by other parasites.

**Fig 1:**
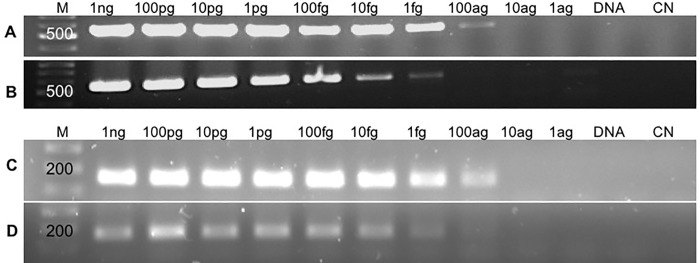
Agarose gel electrophoresis after cPCR assays using HPNF3/R3 primers. (A, B) and HpF3/R3 (C, D) 10-fold serial dilution of plasmids (from 1 ng to 1 ag) and (A, C) the same plasmids with non-infected fan mussel (B, D), DNA: DNA from fan mussel PCR negative; CN: negative control.

The detection limit of *H*. *pinnae* with HPNF3/HPNR3 and HpF3/HpR3 primer pairs has been calculated using the plasmids. Moreover, the same assay was carried out adding DNA of a non-infected fan mussel. The results observed in the **Fig 1** shows that the HPNF3/HPNR3 were able to detect until 100 ag and 1 fg equivalent to 2.07x10^1^ and 2.07x10^2^ copies of SSU rDNA / ng of DNA analysed respectively (**Fig 1A and 1B**). The detection limit for HpF3/HpR3 was equal to 100 ag (30 copies of SSU rDNA / ng of DNA) using plasmid alone and when 100ng DNA of non-infected oyster were added (**Fig 1C and 1D**).

**Fig 2:**
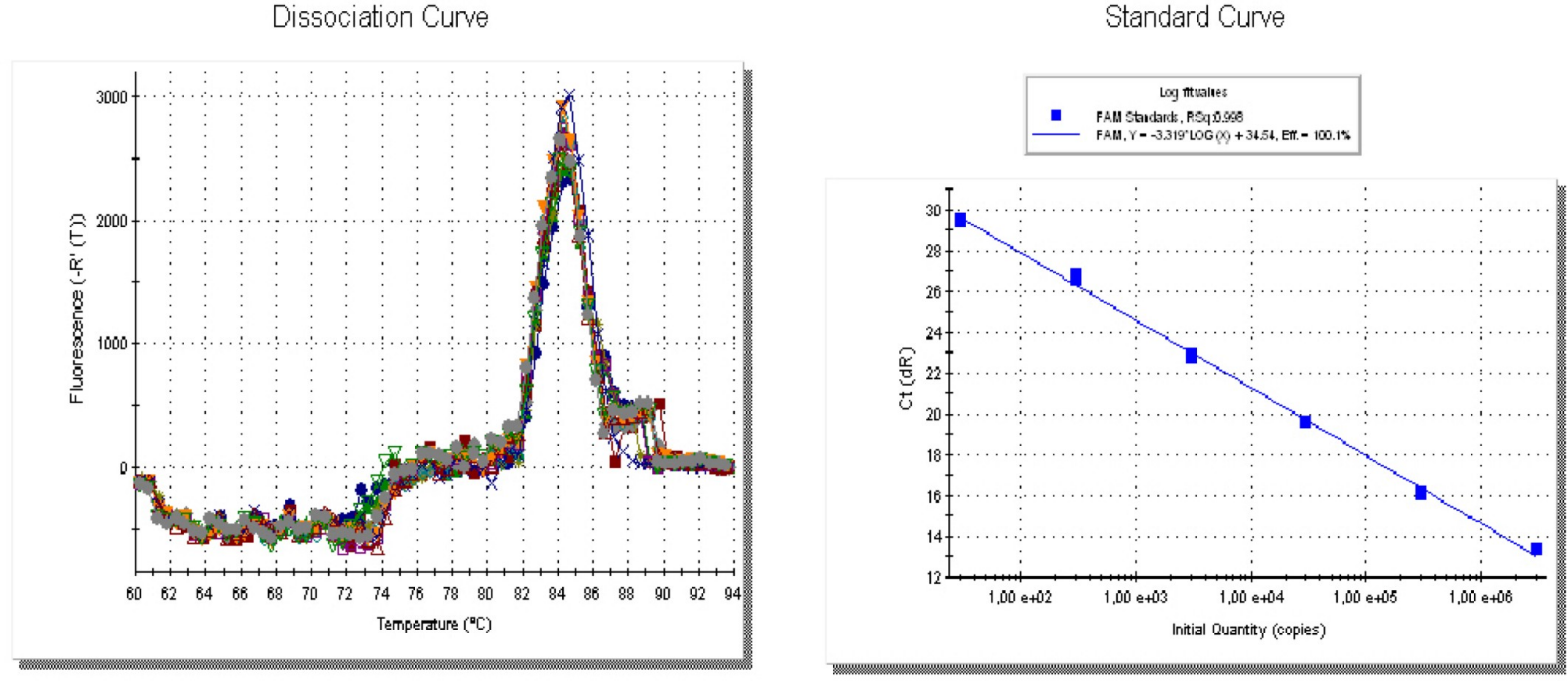
Dissociation curve and melting curve of real-time SYBR Green PCR products of *Haplosporidium pinnae*. (A) Dissociation curve (DC) Melting curve of real-time SYBR Green PCR products of *Haplosporidium pinnae*. A single melt peak at 84°C indicates a single PCR product is being amplified in these samples. (B) Standard curve (SC) covering 10 million to <10 copies of specific *H*. *pinnae* SSU rDNA. E: Efficiency; R^2^: Linearity E = 100.1% R^2^ = 0.998 slope = -3.319 [11].

The qPCR results showed that only one melting temperature peak was observed with a mean of 84.1±0.01°C (**Fig 2**). All sample highlighted a qPCR amplification, except two samples (PN3 and PN7) that resulted negative. Among these, seven samples (PN9, PN23, PN25, PN26, PN27, PN29 and PN30), with no amplification in cPCR, resulted positive using qPCR although some of them resulted with very low number of *H*. *pinnae* SSU rDNA copies (**Table 3**). Excluding two samples (PN25 and PN30) that showed less than 1 copy of SSUrDNA *H*. *pinnae* / ng of fan mussel DNA, results ranged from 6.34E+00 to 7.1E+07. The adductor muscle from PN26-AM was positive for *H*. *pinnae* both by conventional and quantitative PCR.

The limit of detection of the qPCR assay was between 100 to 10 ag which is equivalent of 30 to 3 *H*. *pinnae* SSU rDNA copies / ng of plasmid DNA. The *H*. *pinnae* standard curve displayed an amplification efficiency of 102% and linearity (R^2^) of 0.994. When plasmids with DNA of fan mussel were tested similar results were obtained (E = 103% and R^2^ = 0.992).

The statistical analysis revealed that the number of positive cases for *H*. *pinnae* was significantly higher than the number of negative cases for each assay. The prevalence of *H*. *pinnae* was of 94% (30 / 32) positives or very low number of *H*. *pinnae* SSU rDNA copies versus 6% (2 / 32) negatives according to the qPCR results.

Conventional PCR and qPCR were specific for the molecular diagnosis of *H*. *pinnae* when DNA of other protozoans were used.

## Discussion

The focus of this study was to develop a species-specific quantitative PCR protocol to diagnose *H*. *pinnae*. The SSU rDNA marked proved a good genetic result for both cPCR and qPCR approaches. This gene region is often a useful target for diagnostic tests, because there are both enough variability and many copies in the genome, which help to ensure good specificity and sensitivity [15]. For this reason, in this study the parasitic load of *H*. *pinnae* is reported as copies of *H*. *pinnae* by SSU rDNA / ng of extracted DNA.

The specificity of these assays resulted in PCR amplification of DNA from *H*. *pinnae* only and was negative in all the tests with the DNA of other protozoan parasites. In the last few years, species-specific real-time PCR procedures for the diagnosis of protozoan parasites in different mollusc species have been developed and applied to monitoring protocols. In fact, the protistan parasite *Perkinsus marinus*, a severe pathogen of the oyster *Crassostrea virginica*, was detectable by real-time PCR, showing specificity and sensitivity of this assay [16]. More recently, a rapid and sensitive diagnostic of *Bonamia ostreae* and *B*. *exitiosa* in the European flat oyster *Ostrea edulis* [5] as well as a qPCR assay to determine prevalence and intensity of *Haplosporidium nelsoni* in oysters *Crassostrea virginica* [17], described sensitive techniques that can detect very low quantities of parasite DNA.

The sensitivity of the qPCR technique described in this study using the cloned DNA fragment to avoid inhibitions in PCR reaction (3 SSU rDNA copies of *H*. *pinnae* / ng of fan mussel DNA) is presented as the ideal molecular diagnostic technique for monitoring protocols. Regarding the detection of the SSU rDNA region of *H*. *pinnae*, the recommendation is to use the primers described by Catanese et al 2018 [4] whose sensitivity is 207 copies of the DNA of *H*. *pinnae* / ng of fan mussel DNA.

However, as expected, this study showed some differences in positive amplification resulted using the two methods. In fact, samples negative for cPCR but positive with qPCR confirmed the lower sensitivity of conventional PCR in relation to quantitative approach. Other studies have shown problems with cPCR working with low quantity of tissue, because light infections may be missed if tissue is subsampled or because inhibitory factors in mollusc tissues may give false negative results [15]. In our study, the amplification control supports this hypothesis. In fact, two sample (PN3 and PN7), with negative amplification in qPCR, showed amplicons only for the control amplification in cPCRs but resulting also negative for the *H*. *pinnae* detection. Otherwise in other two samples (PN25 and PN30), qPCR detected <1 copy of *H*. *pinnae* SSU rDNA, but no amplification was observed in cPCR assays, including in the control of amplification, probably due to a strong degradation of the DNA or to the eventual presence of PCR-inhibitors that may not have been eliminated during extraction with the tissue kits.

In this study, the quality of the used sample tissue should be also considered. Although it has been highlighted that the sporulation stages of *H*. *pinnae* were exclusively observed in the digestive gland tubules of *P*. *nobilis*, those resulted often darker and softer than the normal appearance and sometimes presented liquefaction [2–3], indicating that digestive gland is one of the first tissues to degrade after death of fan mussel. The mantle sampling is easy to undertake *in situ* without much disturbance to living specimens and it has been previously tested to obtain samples in many *P*. *nobilis* populations for genetic studies. Moreover, we must consider that these animals are in a critical situation and, in the time of carrying out this study, the authorities only allowed sampling the fan mussels when they were dead, showing evidence of parasite infection. Only at a later time, to increase the monitoring frequency in Western and Central Mediterranean populations, authorities suggested and allowed new precautionary measures, including to take tissue biopsies of the mantle from the alive specimens to find out the presence or absence of the parasite (http://medpan.org/pinna-nobilis-mass-mortality-outbreak-in-the-mediterranean-recommendations-for-mpas). As consequence, the use of the mantle of *P*. *nobilis* as reference tissue for molecular biology *H*. *pinnae* detection was highly recommended. For this study the mantle was sampled and analysed regardless of the presented appearance, although in PN9, PN24, PN25, PN26, PN28, PN29 and PN30 samples showed signs of degradation (ratio 260/280 < 1.8) only PN25 and PN30 failed of cPCR control reaction.

Therefore, we observed that the methods of tissue conservation would seem to have uncommon effects in the cPCR, probably due to the dissimilar action of inhibitors or sensitivity of the different Taq polymerases used by the two laboratories. In contrast, in the qPCR of *H*. *pinnae*, diagnostic was positive for all of them except PN3 and PN7, which did not show DNA degradation signs. Therefore, there are substances in the DNA samples that could inhibit the polymerase of cPCR without affecting the qPCR.

Moreover, we must consider that both laboratories have been using tissue kits for the extraction of the target DNA (Material and methods paragraph “Sample collection and genetic DNA extraction”). Audermard et al 2004 [16] showed that if the sample presents inhibitors, the tissue kit is not efficient to eliminate them. Although in this study, the amount of DNA obtained from each fan mussel has been acceptable, the molecular diagnosis failed and one of the reasons may be that the enzymatic lysis was insufficient due to the poor state of the tissues [18]. Therefore, when the animal to be sampled is dead or has a bad appearance, the recommendation is to use stool kits. On the contrary, if the analysis to be carried out is on living tissues (for example biopsies) the efficiency is significantly higher with tissue kits, especially in bivalve molluscs [16].

The here presented diagnostic protocol by qPCR, revealed that 94% of the 32 analysed mantle tissues of fan mussel were infected by *H*. *pinnae*, showing a high sensitivity and specificity for its detection (100% if we don't consider negative and too much degraded samples). The sensitivity and specificity qPCR method led us to infer that there could be some false positives (very low quantity of copy) or false negative (DNA degradation or presence of PCR-inhibitor compounds).

The application of the assay offers much potential for studies to understand parasite communities and its diffusion, disease risk, host–parasite interactions and parasite impact in ecosystem processes [19]. We do not know how *H*. *pinnae* is transported and infects the fan mussels, even though the most plausible access route of the parasite is the digestive tract during the filtration of nutrients, thus interfering with food absorption and causing severe general dysfunction with fatal outcome [2–3].

The described protocol presents the advantages of high sensitivity and high specificity, and it is reliable for a rapid screening to detect the presence of the pathogen from very small mantle samples, including from the biopsy of alive but potentially infected fan mussels, or to determine the relative abundance in the environment, such as water samples in the field.
