# Correction: Real-Time PCR based test for the early diagnosis of *Haplosporidium pinnae* affecting fan mussel *Pinna nobilis*

**DOI:** 10.1371/journal.pone.0229548

**Published:** 2020-02-18

**Authors:** Monserrat López-Sanmartín, Gaetano Catanese, Amalia Grau, José María Valencia, Jose Rafa García-March, José Ignacio Navas

In Table 2, the sequence of primer HpR3 is incorrect. The correct sequence of primer HpR3 is 5´ AAAACCAACAAAGGCCCGAA 3´. Please see the correct Table 2 here.

**Table 2:** Characteristics of the primers used for PCRs.

Primer	Sequence (5´- 3´)	Fragment size (bp)	Temperature annealing (°C)	Reference
18SFr	CGAGCAATAACAGGTCTGTG	200	50°C	Mauri et al 2011
18SRw	GGCAGGGACTTAATCAA			
HPNF3	CATTAGCATGGAATAATAAAACACGAC	600	55°C	Catanese et al 2018
HPNR3	GCGACGGCTATTTAGATGGCTGA			
HpF	GGTACGGAGAATCCGGGGTT	1409	55 °C	This study
HpR	ACTTGTCCTTCCTCTAATAATAAGG			
HpF3	GCGGGCTTAGTTCAGGGG	165	60 °C	This study
HpR3	AAAACCAACAAAGGCCCGAA			
